# A New Exceptional Family of Elements and Solvability of General Order Complementarity Problems

**DOI:** 10.1155/2014/248462

**Published:** 2014-05-22

**Authors:** Na Huang, Changfeng Ma

**Affiliations:** School of Mathematics and Computer Science, Fujian Normal University, Fuzhou 350007, China

## Abstract

By using the concept of exceptional family, we propose a sufficient condition of a solution to general order complementarity problems (denoted by GOCP) in Banach space, which is weaker than that in Németh, 2010 (Theorem 3.1). Then we study some sufficient conditions for the nonexistence of exceptional family for GOCP in Hilbert space. Moreover, we prove that without exceptional family is a sufficient and necessary condition for the solvability of pseudomonotone general order complementarity problems.

## 1. Introduction

There are several types of order complementarity problems in real world applications. Among them, the linear order complementarity problem was systematically studied (see [1]). The problem was extended to the general linear order complementarity problem and some interesting results have been presented (see [2–4]). In [4], Sznajder extended the linear order complementarity problem to the nonlinear order complementarity problem. The notion of the general order complementarity problem considered in this paper is taken from [3, 5, 6].

There are many problems in engineering, management science, and other fields which can be reformulated as general order complementarity problems. But we are interested in the solvability of the problem. The concept of exceptional family is a powerful tool to study existence theorems of the solution to nonlinear complementarity problems and variational inequality problems (see [7–15]). Smith first introduced in [16] the notion of exceptional sequence of elements for continuous functions in order to investigate the solution existence of nonlinear complementarity problems. In 1997, Zhao first extended the concept of exceptional family for variational inequalities (see [17]). Several years later, Isac and Zhao extended the concept of exceptional family to variational inequalities in *R* ^*n*^ to general Hilbert space (see [18]). Using the more general notion of exceptional family of elements introduced by Isac et al. (see [19]) and Kalashnikov (see [20]), some existence theorems for complementarity problems are presented (see [19, 21]). In 2008, Zhang proposed an existence theorem for semidefinite complementarity problem (denoted by SDCP). He introduced generalizations of Isac-Carbone's condition and proved that Isac-Carbone's condition is the sufficient conditions for the solvability of SDCP (see [22]). In 2012, Hu et al. proposed an existence theorem for copositive complementarity problem (denoted by CCP) and extended the property of coercivity, *p*-order coercivity, monotone, and (strictly) weakly proper to CCP (see [23]). In 2010, Németh first introduced the notion of exceptional family for general order complementarity problems in Banach space and used the notion to study the solvability of general order complementarity problems (see [6]).

Motivated and inspired by the works mentioned above, in this paper, by using the concept of exceptional family in [6], we propose a sufficient condition of a solution to general order complementarity problems (denoted by GOCP) in Banach space, which is weaker than that in [6, Theorem  3.1]. Then we study some sufficient conditions for the nonexistence of exceptional family for GOCP in Hilbert space. Moreover, we prove that the nonexistence of exceptional family is a sufficient and necessary condition for the solvability of pseudomonotone general order complementarity problems.

The remainder of this paper is organized as follows. The preliminary results which will be used in this paper are stated in Section 2. In Section 3, we recall the definition of general order complementarity problems (see [3, 5, 6]) and introduced the concept of exceptional family for the general order complementarity problems (see [6]), then we prove an essential result. In Section 4, we discuss the conditions for the nonexistence of exceptional family. Conclusions are drawn in Section 5.

## 2. Preliminaries

In this section, we recall some background materials and preliminary results used in the subsequent sections. Firstly, we give some concepts from [6].

Let *X* be a Banach space whose norm is denoted by ||·||. Let *K* ⊂ *H* be a closed set. *K* is called a wedge, if for any *λ* ≥ 0 and *x*, *y* ∈ *K*, *λx* ∈ *K* and *x* + *y* ∈ *K*. A wedge *K* is called a cone if *K*∩(−*K*) = {0}.

**Definition 1 (see [6])**

A relation ⪯ on *X* is called an order if it meets

1. reflexivity; that is, *x*⪯*x* for all *x* ∈ *X*;
2. antisymmetry; that is, if *x*⪯*y* and *y*⪯*x*, then *x* = *y*;
3. transitivity; that is, if *x*⪯*y* and *y*⪯*z*, then *x*⪯*z*.

We say a relation ⪯ on *X* is induced by a cone *K* ⊂ *X*; that is, *x*⪯*y* if and only if *y* − *x* ∈ *K*. Hence *K* = {*x* ∈ *X* : 0⪯*x*} by using the relation ⪯ on *X*. Then we denote an ordered Banach space by (*X*, ||·||, *K*).

**Property 1 (see [6])**

A relation ⪯ on *X* is induced by a cone *K* ⊂ *X* if and only if it is

1. translation invariant; that is, if *x*⪯*y*, then *x* + *z*⪯*y* + *z* for all *z* ∈ *X*;
2. scale invariant; that is, if *x*⪯*y*, then *λx*⪯*λy* for any *λ* > 0;
3. continuous; that is, if for any two convergent sequences {*x*_*n*_}_*n*>0_ and {*y*_*n*_}_*n*>0_ in *X* with *x*_*n*_⪯*y*_*n*_ for all *n* > 0, then x*⪯y*, where x* and y* are the limits of {*x*_*n*_}_*n*>0_ and {*y*_*n*_}_*n*>0_, respectively.

The ordered Banach space (*X*, ||·||, *K*) is called a vector lattice if for every *x*, *y* ∈ *X* there exists *x*∧*y* : = inf⁡{*x*, *y*} with respect to the order induced by *K*. In this case we say that the cone *K* is latticial. By the above concepts, we give the following property from [6].

**Property 2**

Let (*X*, ||·||) be a Banach space ordered by the latticial cone *K* ⊂ *X*. For each *x* ∈ *X* we denote *x*_+_ = −0∧(−*x*); then the following two equalities hold for all *x*, *y*, *z* ∈ *X*:

1. (*x* + *z*)∧(*y* + *z*) = *x*∧*y* + *z*;
2. *x*∧*y* = *y* − (*y* − *x*)_+_.

A continuous mapping *F* : *Ω*⊆*X* → *X* is called completely continuous mapping if for every bounded set Δ⊆*Ω* the set *F*(Δ) is relatively compact. The notation deg(*F*, *Ω*, *y*) is the topological degree associated with *F*, *Ω*, and *y* (see [24, 25]). Now we recall briefly the notation and some key properties of topological degree that will be used below.

**Theorem 2 (see [8, Theorem 1.1])**

Let *Ω*⊆*X* be an open bounded subset, and let *I* : *X* → *X* be an identity mapping; that is, *I*(*x*) = *x*, ∀*x* ∈ *X*. Then deg⁡(*I*, *Ω*, *p*) = 1, ∀*p* ∈ *Ω*.

**Theorem 3 (Poincaré-Bohl theorem, see [26])**

Let *Ω*⊆*X* be an open bounded subset, and let *H* : [0,1] × *Ω* → *X* be a completely continuous mapping. If *y* ∉ *h*_*t*_(∂*Ω*), then deg⁡(*h*_*t*_, *Ω*, *y*) is a constant for 0 ≤ *t* ≤ 1, where *h*_*t*_(*x*) = *x* − *H*(*t*, *x*).

**Theorem 4 (Kronecker theorem, see [27])**

Let *Ω*⊆*X* be an open bounded subset, *I* : *X* → *X* an identity mapping, and f  : $\overset{-}{\Omega }\to X$ a completely continuous mapping. If deg⁡(*I* − *f*, *Ω*, *y*) ≠ 0, then equation *x* − *f*(*x*) = *y* has at least one solution in *Ω*.

## 3. Exceptional Family for GOCP

First we recall the definition of general order complementarity problems (see [3, 5, 6]) and next we recall the concept of exceptional family for general order complementarity problems (GOCPs) (see [6]).

**Definition 5**

Let (*X*, ||·||) be a Banach space ordered by the latticial cone *K* ⊂ *X* and *D* ⊂ *X* a nonempty closed convex set. Consider *m* mappings *f*_1_, *f*_2_,…, *f*_*m*_ : *X* → *X*. The general order complementarity problem defined by the family of mappings {*f*_*i*_}_*i*=1_^*m*^ and the set *D* is

(1) $$\begin{array}{c} \text{GOCP}\left({\left\{f_{i}\right\}}_{i=1}^{m},D\right):\{\begin{array}{cc} \text{find}\;\;x^{*}\in D\;\text{such}\;\text{that} & \\ f_{1}\left(x^{*}\right)\wedge \cdots \wedge f_{m}\left(x^{*}\right)=0. & \end{array} \end{array}$$

**Definition 6**

Let (*X*, ||·||) be a Banach space ordered by the latticial cone *K* ⊂ *X* and *D* ⊂ *X* a nonempty closed convex set. Consider *m* mappings *f*_1_, *f*_2_,…, *f*_*m*_ : *X* → *X*. A sequence {*x* ^*r*^}_*r*>0_⊆*D* is said to be an exceptional family for GOCP({*f*_*i*_}_*i*=1_^*m*^, *D*) if the following conditions are satisfied:

1. ||*x*_*r*_|| → +*∞* as *r* → +*∞*,
2. for every real number *r* > 0, there exists a real number *μ*_*r*_ > 0 such that *u*_*r*_^1^∧⋯∧*u*_*r*_^*m*^ = 0, with *u*_*r*_^*i*^ = *μ*_*r*_ *x*_*r*_ + *f*_*i*_(*x*_*r*_), for *i* = 1,2,…, *m*.

The following lemma comes from the property proved in [6, Theorem  3.1]. Here we recall the property and the proof which are the same as the proof in [6, Theorem  3.1].

**Lemma 7**

Let *ϕ*(*x*) = *x* − *f*_1_(*x*)∧⋯∧*f*_*m*_(*x*). If *f*_*i*_ and *S*_*i*_ = *I* − *f*_*i*_ are completely continuous for all *i* = 1,2,…, *m*, so is *ϕ*(*x*).

**Proof**

Let

(2) $$\begin{array}{c} \phi_{k}\left(x\right):=x-f_{1}\left(x\right)\wedge \cdots \wedge f_{k}\left(x\right), \end{array}$$

for all *x* ∈ *H* and *k* = 1,2,…, *m*. We will prove by induction that *ϕ*_*k*_ are completely continuous mappings for all *k* = 1,2,…, *m*. If *k* = 1, then *ϕ*_1_(*x*) = *x* − *f*_1_(*x*) = *S*_1_(*x*). From the condition we see that *ϕ*_1_ is a completely continuous mapping immediately. Suppose that *ϕ*_*k*_ is a completely continuous mapping where *k* ∈ {1,2,…, *m* − 1}. By (2), the definition of *S*_*k*+1_(*x*), and Property 2(2), we have

(3) ϕk+1(x)=x−f1(x)∧⋯∧fk(x)∧fk+1(x)=x−(x−ϕk(x))∧fk+1(x)=x−(x−ϕk(x))∧(x−Sk+1(x))=x−[(x−Sk+1(x))−(x−Sk+1(x)−x+ϕk(x))+]=Sk+1(x)+(ϕk(x)−Sk+1(x))+.

Hence, *ϕ*_*k*_ are completely continuous mappings, for all *k* = 1,2,…, *m*. In particular, *ϕ*(*x*) = *ϕ*_*m*_(*x*) is a completely continuous mapping.

In what follows, we will establish an important theorem for GOCP({*f*_*i*_}_*i*=1_^*m*^, *D*).

**Theorem 8**

Let (*X*, ||·||) be a Banach space ordered by the latticial cone *K* ⊂ *X* and *D* ⊂ *X* an unbounded closed convex set. If *f*_*i*_ and *S*_*i*_ = *I* − *f*_*i*_ are completely continuous for all *i* = 1,2,…, *m*, then GOCP({*f*_*i*_}_*i*=1_^*m*^, *D*) has either a solution or an exceptional family.

**Proof**

From Definition 5 we know that the solvability of the problem GOCP is equivalent to the problem of finding an *x* ∈ *D* such that *ϕ*(*x*) = *x*. Let *H*(*t*, *x*) = (1 − *t*)*ϕ*(*x*), 0 ≤ *t* ≤ 1. We get that *H*(*t*, *x*) is completely continuous mapping from Lemma 7. Consider a family of spheres *B*_*r*_ and open balls *U*_*r*_:

(4) $$\begin{array}{c} B_{r}=\left\{x\in D:||x||=r\right\},\;\;U_{r}=\left\{x\in D:||x||<r\right\}. \end{array}$$

Since *D* is unbounded, we have *B*_*r*_ ≠ *∅* and *U*_*r*_ ≠ *∅* for all *r* > 0. We consider the mapping *h*_*t*_(*x*) = *x* − *H*(*t*, *x*), *t* ∈ [0,1]. If there exists an *r* > 0 such that

(5) $$\begin{array}{c} 0\notin \left\{h_{t}\left(x\right):x\in B_{r},t\in \left[0,1\right]\right\}. \end{array}$$

It follows from Theorem 3 that deg(*h*_*t*_(*x*), *U*_*r*_, 0) is constant for *t* ∈ [0,1]. This together with Theorem 2 implies that deg(*x* − *ϕ*(*x*), *U*_*r*_, 0) = deg⁡(*x*, *U*_*r*_, 0) = 1. Therefore, we know that problem *ϕ*(*x*) = *x* is solvable from Theorem 4; that is, the problem GOCP is solvable.

On the other hand, for every *r* > 0, there exist a vector *x*_*r*_ ∈ *B*_*r*_ and a scalar *t*_*r*_ ∈ [0,1] such that *h*_*t*_*r*__(*x*_*r*_) = 0; that is, *x*_*r*_ − (1 − *t*_*r*_)*ϕ*(*x*_*r*_) = 0.

If *t*_*r*_ = 0, then *x*_*r*_ − *ϕ*(*x*_*r*_) = 0, which again implies solvability of the problem GOCP. If *t*_*r*_ = 1, then *x* ^*r*^ = 0, which contradict with the fact *x*_*r*_ ∈ *B*_*r*_. Hence *t*_*r*_ ≠ 1. If 0 < *t*_*r*_ < 1, then from the definition of *ϕ*(*x*) we get

(6) $$\begin{array}{c} x_{r}-\left(1-t_{r}\right)\left(x_{r}-f_{1}\left(x_{r}\right)\wedge \cdots \wedge f_{m}\left(x_{r}\right)\right)=0; \end{array}$$

that is,

(7) $$\begin{array}{c} t_{r}x_{r}+\left(1-t_{r}\right)f_{1}\left(x_{r}\right)\wedge \cdots \wedge f_{m}\left(x_{r}\right)=0. \end{array}$$

Dividing both parts by 1 − *t*_*r*_, we obtain

(8) $$\begin{array}{c} \mu_{r}x_{r}+f_{1}\left(x_{r}\right)\wedge \cdots \wedge f_{m}\left(x_{r}\right)=0, \end{array}$$

where *μ*_*r*_ = *t*_*r*_/(1 − *t*_*r*_). Let *u*_*r*_^*i*^ = *μ*_*r*_ *x*_*r*_ + *f*_*i*_(*x*_*r*_), *i* = 1,2,…, *m*; then

(9) ur1∧⋯∧urm=(μrxr+f1(xr))∧⋯∧(μrxr+fm(xr))=μrxr+f1(xr)∧⋯∧fm(xr)=0,

where the second equality follows from Property 2(1). Thus, {*x*_*r*_}_*r*>0_ is an exceptional family for GOCP. The proof is complete.

**Remark 9**

Notice that, in [6], they used the condition of completely continuous field instead of *f*_*i*_ and *S*_*i*_ = *I* − *f*_*i*_ being completely continuous operators for all *i* = 1,2,…, *m*. Moreover, [6, Theorem  3.1] required that the condition *S*_*m*_(*D*) + *K* ⊂ *D* holds, which does not need this condition in Theorem 8 in our paper. Hence, our condition in Theorem 8 is weaker than the condition of [6, Theorem  3.1].

## 4. Existence Conditions of a Solution to GOCP

In this section, we consider the general order complementarity problems in Hilbert space *H* whose inner product and norm are denoted by 〈·, ·〉 and ||·||, respectively. We propose some sufficient conditions and prove that they guarantee existence of solutions to the general order complementarity problem. Firstly, we give the condition as follows.

**Condition 1**

Let (*H*, 〈·, ·〉) be a Hilbert space ordered by the latticial cone *K* ⊂ *H* and *D* ⊂ *H* a nonempty set. *f*_1_, *f*_2_,…, *f*_*m*_ : *H* → *H* satisfy the following condition: there exists *ρ* > 0 such that for all *x* ∈ *D* with ||*x*|| > *ρ*, there exists *y* ∈ *H* with ||*y*|| < ||*x*|| such that

(10) $$\begin{array}{c} \langle x-y,f_{1}\left(x\right)\wedge \cdots \wedge f_{m}\left(x\right)\rangle \geq 0. \end{array}$$

**Theorem 10**

Let (*H*, 〈·, ·〉) be a Hilbert space ordered by the latticial cone *K* ⊂ *H*, *D* ⊂ *H* an unbounded closed convex set and *f*_*i*_, *S*_*i*_ = *I* − *f*_*i*_ are completely continuous for all *i* = 1,2,…, *m*. If Condition 1 holds, then there exists no exceptional family for GOCP({*f*_*i*_}_*i*=1_^*m*^, *D*) and hence, GOCP({*f*_*i*_}_*i*=1_^*m*^, *D*) is solvable.

**Proof**

Suppose that GOCP({*f*_*i*_}_*i*=1_^*m*^, *D*) has an exceptional family {*x*_*r*_}_*r*>0_ ⊂ *D*. By Definition 6, we have

(11) $$\begin{array}{c} u_{r}^{i}=\mu_{r}x_{r}+f_{i}\left(x_{r}\right),\;\;\mu_{r}>0,\;\;i=1,2,\ldots ,m\;\;\left(\forall r>0\right),\\ u_{r}^{1}\wedge \cdots \wedge u_{r}^{m}=0\;\left(\forall r>0\right),\\ ||x_{r}||⟶+\infty \;\text{as}\;r⟶+\infty . \end{array}$$

Take *r* > 0 such that ||*x*_*r*_|| > *ρ*. Since *f*_1_, *f*_2_,…, *f*_*m*_ satisfy Condition 1, there exists *y*_*r*_ ∈ *H* with ||*y*_*r*_|| < ||*x*_*r*_|| such that 〈*x*_*r*_ − *y*_*r*_, *f*_1_(*x*_*r*_)∧⋯∧*f*_*m*_(*x*_*r*_)〉≥0. We have

(12) 0≤〈xr−yr,f1(xr)∧⋯∧fm(xr)〉=〈xr−yr,−μrxr〉=−μr||xr||2+μr〈yr,xr〉≤−μr||xr||2+μr||yr||||xr||=−μr||xr||(||xr||−||yr||)<0,

which is impossible. Hence, there exists no exceptional family for GOCP({*f*_*i*_}_*i*=1_^*m*^, *D*). Then the problem is solvable.

**Condition 2**

Let (*H*, 〈·, ·〉) be a Hilbert space ordered by the latticial cone *K* ⊂ *H* and *D* ⊂ *H* a nonempty set. *f*_1_, *f*_2_,…, *f*_*m*_ : *H* → *H* satisfy the following condition: there exists a nonempty bounded subset *C* ⊂ *D* such that for every *x* ∈ *D*∖*C*, there exists *y* ∈ *C* such that

(13) $$\begin{array}{c} \langle x-y,f_{1}\left(x\right)\wedge \cdots \wedge f_{m}\left(x\right)\rangle \geq 0. \end{array}$$

**Corollary 11**

Let (*H*, 〈·, ·〉) be a Hilbert space ordered by the latticial cone *K* ⊂ *H* and *D* ⊂ *H* an unbounded closed convex set and *f*_*i*_, *S*_*i*_ = *I* − *f*_*i*_ are completely continuous for all *i* = 1,2,…, *m*. If Condition 2 holds, then there exists no exceptional family for GOCP({*f*_*i*_}_*i*=1_^*m*^, *D*) and hence, GOCP({*f*_*i*_}_*i*=1_^*m*^, *D*) is solvable.

**Proof**

Let *C* ⊂ *D* be the set defined by Condition 2. Since *C* is bounded, then there exists *ρ* > 0 such that *C* ⊂ *U*_*ρ*_∩*D*, where *U*_*ρ*_ = {*x* ∈ *H* : ||*x*||≤*ρ*}. For any *x* such that ||*x*|| > *ρ*, there exists *y* ∈ *C*  (||*y*|| ≤ *ρ* < ||*x*||) such that 〈*x* − *y*, *f*_1_(*x*)∧⋯∧*f*_*m*_(*x*)〉≥0. Hence Condition 1 is satisfied. This together with Theorem 10 completes the proof.

We extend the coercivity condition and *p*-order coercivity condition (see [15, 23]) to GOCP as follows.

**Definition 12**

Let (*H*, 〈·, ·〉) be a Hilbert space ordered by the latticial cone *K* ⊂ *H* and *D* ⊂ *H* an unbounded set. Consider *m* mappings *f*_1_, *f*_2_,…, *f*_*m*_ : *H* → *H*. *f*_1_(*x*)∧⋯∧*f*_*m*_(*x*) is said to be *p*-order coercive with respect to *D*, if there exists *p* ∈ (−*∞*, 1] and *y* ∈ *H* such that

(14) $$\begin{array}{c} \underset{x\in D,||x||\to +\infty }{\lim}\frac{\langle x-y,f_{1}\left(x\right)\wedge \cdots \wedge f_{m}\left(x\right)\rangle }{{||x||}^{p}}=+\infty . \end{array}$$

**Theorem 13**

Let (*H*, 〈·, ·〉) be a Hilbert space ordered by the latticial cone *K* ⊂ *H* and *D* ⊂ *H* an unbounded closed convex set and *f*_*i*_, *S*_*i*_ = *I* − *f*_*i*_ are completely continuous for all *i* = 1,2,…, *m*. If there exists some *p* ∈ (−*∞*, 1] such that *f*_1_(*x*)∧⋯∧*f*_*m*_(*x*) is *p*-order coercive with respect to *D*, then there exists no exceptional family for GOCP({*f*_*i*_}_*i*=1_^*m*^, *D*) and hence, GOCP({*f*_*i*_}_*i*=1_^*m*^, *D*) is solvable.

**Proof**

Since *f*_1_(*x*)∧⋯∧*f*_*m*_(*x*) is *p*-order coercive with respect to *D* for some *p* ∈ (−*∞*, 1], we get

(15) $$\begin{array}{c} \underset{x\in D,||x||\to +\infty }{\lim}\frac{\langle x-y,f_{1}\left(x\right)\wedge \cdots \wedge f_{m}\left(x\right)\rangle }{{||x||}^{p}}=+\infty . \end{array}$$

This together with ||*x*||^*p*^ ≥ 0 and the definition of an infinite limit yields that

(16) $$\begin{array}{c} \langle x-y,f_{1}\left(x\right)\wedge \cdots \wedge f_{m}\left(x\right)\rangle \geq 0, \end{array}$$

for sufficiently large *ρ* and ||*x*|| > *ρ*. Hence Condition 1 is satisfied. This together with Theorem 10 completes the proof.

The following results extend monotone property and (strictly) weakly proper to GOCP.

**Definition 14**

Let (*H*, 〈·, ·〉) be a Hilbert space ordered by the latticial cone *K* ⊂ *H* and *D* ⊂ *H* a nonempty set. Consider *m* mappings *f*_1_, *f*_2_,…, *f*_*m*_ : *H* → *H*. *f*_1_(*x*)∧⋯∧*f*_*m*_(*x*) is said to be

(a) pseudomonotone on *D* if, for every *x*, *y* ∈ *D*, *x* ≠ *y*, one has
  (17) 〈y−x,f1(x)∧⋯∧fm(x)〉 ≥0⟹〈y−x,f1(y)∧⋯∧fm(y)〉≥0;
(b) quasiomonotone on *D* if, for every *x*, *y* ∈ *D*, *x* ≠ *y*, one has
  (18) 〈y−x,f1(x)∧⋯∧fm(x)〉 >0⟹〈y−x,f1(y)∧⋯∧fm(y)〉≥0.

**Definition 15**

Let (*H*, 〈·, ·〉) be a Hilbert space ordered by the latticial cone *K* ⊂ *H* and *D* ⊂ *H* an unbounded set. Consider *m* mappings *f*_1_, *f*_2_,…, *f*_*m*_ : *H* → *H*. *f*_1_(*x*)∧⋯∧*f*_*m*_(*x*) is said to be

(a) weakly proper on *D*, if for every sequence {*x*_*r*_} ⊂ *D* with lim⁡_*r*→+*∞*_||*x*_*r*_|| = +*∞*, there exists a *y* ∈ *H* and some *r* such that
  (19) $$\begin{array}{c} \langle x_{r}-y,f_{1}\left(y\right)\wedge \cdots \wedge f_{m}\left(y\right)\rangle \geq 0,\;||x_{r}||>||y||; \end{array}$$
(b) strictly weakly proper on *D*, if for every sequence {*x*_*r*_} ⊂ *D* with lim⁡_*r*→+*∞*_||*x*_*r*_|| = +*∞*, there exists a *y* ∈ *H* and some *r* such that
  (20) $$\begin{array}{c} \langle x_{r}-y,f_{1}\left(y\right)\wedge \cdots \wedge f_{m}\left(y\right)\rangle >0,\;||x_{r}||>||y||. \end{array}$$

**Theorem 16**

Let (*H*, 〈·, ·〉) be a Hilbert space ordered by the latticial cone *K* ⊂ *H* and *D* ⊂ *H* an unbounded closed convex set and *f*_*i*_, *S*_*i*_ = *I* − *f*_*i*_ are completely continuous for all *i* = 1,2,…, *m*. If *f*_1_(*x*)∧⋯∧*f*_*m*_(*x*) is pseudomonotone on *D*, then the following conditions are equivalent:

1. GOCP({*f*_*i*_}_*i*=1_^*m*^, *D*) has no exceptional family;
2. GOCP({*f*_*i*_}_*i*=1_^*m*^, *D*) has at least a solution;
3. *f*_1_(*x*)∧⋯∧*f*_*m*_(*x*) is weakly proper on *D*.

**Proof**

(1)⇒(2) follows from Theorem 13.

(2)⇒(3). Since GOCP({*f*_*i*_}_*i*=1_^*m*^, *D*) has at least a solution, there exists x* ∈ *D* such that *f*_1_(x*)∧⋯∧*f*_*m*_(x*) = 0. Then for every sequence {*x*_*r*_} ⊂ *D* with lim⁡_*r*→+*∞*_||*x*_*r*_|| = +*∞*, we have

(21) $$\begin{array}{c} \langle x_{r}-x^{*},f_{1}\left(x^{*}\right)\wedge \cdots \wedge f_{m}\left(x^{*}\right)\rangle =0, \end{array}$$

which implies that *f*_1_(*x*)∧⋯∧*f*_*m*_(*x*) is weakly proper on *D*.

(3)⇒(1). Suppose that GOCP({*f*_*i*_}_*i*=1_^*m*^, *D*) has an exceptional family. Then there exists {*x* ^*r*^}_*r*>0_⊆*D*, ||*x*_*r*_|| → +*∞*  (*r* → +*∞*), and *μ*_*r*_ > 0 such that

(22) $$\begin{array}{c} u_{r}^{i}=\mu_{r}x_{r}+f_{i}\left(x_{r}\right),\;i=1,2\ldots ,m,\\ u_{r}^{1}\wedge \cdots \wedge u_{r}^{m}=0. \end{array}$$

From Property 2(1), we obtain

(23) ur1∧⋯∧urm=(μrxr+f1(xr))∧⋯∧(μrxr+fm(xr))=μrxr+f1(xr)∧⋯∧fm(xr)=0;

namely,

(24) $$\begin{array}{c} f_{1}\left(x_{r}\right)\wedge \cdots \wedge f_{m}\left(x_{r}\right)=-\mu_{r}x_{r}. \end{array}$$

Since *f*_1_(*x*)∧⋯∧*f*_*m*_(*x*) is weakly proper on *D*, then there exists a *y* ∈ *H* and some *r* such that

(25) $$\begin{array}{c} \langle x_{r}-y,f_{1}\left(y\right)\wedge \cdots \wedge f_{m}\left(y\right)\rangle \geq 0,\;||x_{r}||>||y||. \end{array}$$

This together with the fact that *f*_1_(*x*)∧⋯∧*f*_*m*_(*x*) is pseudomonotone on *D* yields

(26) $$\begin{array}{c} \langle x_{r}-y,f_{1}\left(x_{r}\right)\wedge \cdots \wedge f_{m}\left(x_{r}\right)\rangle \geq 0. \end{array}$$

By (24) we get

(27) 0≤〈xr−y,f1(xr)∧⋯∧fm(xr)〉=〈xr−y,−μrxr〉=−μr||xr||2+μr〈yr,xr〉≤−μr||xr||2+μr||yr||||xr||=−μr||xr||(||xr||−||yr||)<0,

which is impossible. Hence GOCP({*f*_*i*_}_*i*=1_^*m*^, *D*) has no exceptional family. From the above, we complete the proof.

**Remark 17**

The above theorem shows that if *f*_1_(*x*)∧⋯∧*f*_*m*_(*x*) is pseudomonotone on *D*, GOCP({*f*_*i*_}_*i*=1_^*m*^, *D*) has no exceptional family ⇔ GOCP({*f*_*i*_}_*i*=1_^*m*^, *D*) which has at least a solution.

**Theorem 18**

Let (*H*, 〈·, ·〉) be a Hilbert space ordered by the latticial cone *K* ⊂ *H* and *D* ⊂ *H* an unbounded closed convex set and *f*_*i*_, *S*_*i*_ = *I* − *f*_*i*_ are completely continuous for all *i* = 1,2,…, *m*. If *f*_1_(*x*)∧⋯∧*f*_*m*_(*x*) is quasimonotone on *D*, then there exists no exceptional family for GOCP({*f*_*i*_}_*i*=1_^*m*^, *D*) and hence, GOCP({*f*_*i*_}_*i*=1_^*m*^, *D*) is solvable.

**Proof**

Suppose that GOCP({*f*_*i*_}_*i*=1_^*m*^, *D*) has an exceptional family. Then from the proof of Theorem 16 we obtain (24). Since *f*_1_(*x*)∧⋯∧*f*_*m*_(*x*) is strictly weakly proper on *D*, then there exists a *y* ∈ *H* and some *r* such that

(28) $$\begin{array}{c} \langle x_{r}-y,f_{1}\left(y\right)\wedge \cdots \wedge f_{m}\left(y\right)\rangle >0,\;||x_{r}||>||y||. \end{array}$$

This together with the fact that *f*_1_(*x*)∧⋯∧*f*_*m*_(*x*) is quasimonotone on *D* yields

(29) $$\begin{array}{c} \langle x_{r}-y,f_{1}\left(x_{r}\right)\wedge \cdots \wedge f_{m}\left(x_{r}\right)\rangle \geq 0. \end{array}$$

By (24) we get

(30) 0≤〈xr−y,f1(xr)∧⋯∧fm(xr)〉=〈xr−y,−μrxr〉=−μr||xr||2+μr〈yr,xr〉≤−μr||xr||2+μr||yr||||xr||=−μr||xr||(||xr||−||yr||)<0,

which is impossible. Hence GOCP({*f*_*i*_}_*i*=1_^*m*^, *D*) has no exceptional family. From the above, we complete the proof.

## 5. Conclusion

In this paper, by using the concept of exceptional family in [6], we propose an existence theorem of a solution to general order complementarity problems in Banach space. Then we study some sufficient conditions for the nonexistence of exceptional family in Hilbert space. Moreover, we prove that nonexistence of exceptional family is a sufficient and necessary condition for the solvability of pseudomonotone general order complementarity problems.
